# A Comprehensive Review of Digital Twin from the Perspective of Total Process: Data, Models, Networks and Applications

**DOI:** 10.3390/s23198306

**Published:** 2023-10-08

**Authors:** Honghai Wu, Pengwei Ji, Huahong Ma, Ling Xing

**Affiliations:** The School of Information Engineering, Henan University of Science and Technology, Luoyang 471023, China; honghai2018@haust.edu.cn (H.W.); jipengwei05@163.com (P.J.); mhh@haust.edu.cn (H.M.)

**Keywords:** digital twin, twin data, twin model, twin network, twin deployment

## Abstract

With the rapid development of industrial digitalization and intelligence, there is an urgent need to accurately depict the physical world in digital space, and, in turn, regulate and optimize the behavior of physical entities based on massive data collection and analysis. As a technology that combines virtual space and physical space, digital twin can satisfy all of the above needs, and has attracted widespread attention. Due to the promising application prospects of digital twins, both academia and industry have launched research in this field, and related studies have been conducted from different perspectives. Accordingly, some articles summarizing the existing work have also been published, but they are all from a single perspective, lacking a systematic introduction and summary. Based on this, this paper conducts a comprehensive review of the existing work on digital twins from four perspectives: data, model, network and application, and strives to gain a better understanding of the development of the field from the physical to the virtual and back to the physical. Meanwhile, current research challenges and future directions for the development of digital twins are all discussed.

## 1. Introduction

With the rapid development of industrial digitalisation, informatization and intelligence, information interaction between physical and virtual spaces has become increasingly important and frequent in various industries and fields. It has become an urgent need to achieve the description of physical space in information space, as well as to complete the accurate prediction of physical space evolution and the accurate management of physical entity behaviour based on enormous data collection and analysis. Furthermore, the emergence of next-generation technologies such as the Internet of Things (IoT), artificial intelligence (AI), next-generation mobile communications (6G), big data (BD), virtual reality (VR) and augmented reality (AR) has resulted in significant advancements in simulation, data acquisition, data communication, computation and analysis [1]. This is fueling the rapid expansion of this demand across a range of sectors, encompassing smart manufacturing, intelligent transportation, aerospace and smart cities [2]. As a result, researchers have been urgently searching for a new technology to meet this demand. The digital twin, which can sense, diagnose and predict the state of physical entites in real time, regulate the behaviour of physical entites through collaboration and optimization decision making between twins, realize self evolution through self-learning across relevant digital models, and achieve the autonomous intelligent evolution of total twin networks, is regarded as the most promising way to satisfy the above requirements.

The concept of digital twins has been proposed for over twenty years, but more in-depth discussion, research and application on the related technologies of digital twins have only attracted attention in recent years. Professor Grieves initially introduced Digital Twin (DT) technology in a Product Lifecycle Management (PLM) course in 2002, and since then the meaning of the Digital Twin has been debated many times by researchers from different disciplines. A digital twin, according to Kritzinger et al. [3], is a digital representation of a physical entity with a fully integrated and automated bi-directional data flow to the physical entity. Wu et al. [4] separated the digital twin into three parts: a physical entity and its virtual counterpart, as well as a mapping between the actual object and the virtual twin, allowing the physical and virtual sides to co-evolve. Tao et al. [5] defined digital twins as a PLM component that uses physical, virtual and interactive data from the product lifecycle to map products in real time. Zhuang et al. [6] defined a digital twin as a virtual dynamic model in a virtual environment that exactly replicates a physical entity in the actual world. Barricelli et al. [7] considers a DT as a living, intelligent and evolving model, serving as the virtual counterpart of a physical entity or process. These different definitions provide multiple perspectives on digital twins, demonstrating the wide range of attention to digital twins in research and practice.

With the rapid development of the field of digital twins, more and more scholars have devoted themselves to the theoretical and practical research related to digital twins. The conceptual affinity between digital shadows and digital twins has prompted extensive research efforts, albeit not without its share of perplexing questions. Nevertheless, it is important to emphasize that digital twins and digital shadows have different natures and play different roles in the interaction between the physical and the virtual. As previously mentioned, digital twins encompass a fully integrated and automated bi-directional data flow between physical entities and their digital counterparts. In contrast, digital shadows primarily focus on representing the state of physical objects in the digital realm, lacking the same level of interactivity and autonomous evolution as digital twins [8]. Moreover, according to Bergs et al. [9], digital shadows can be regarded as a forerunner to digital twins, representing a digital depiction of the state of a specific physical entity at a particular moment in time. Digital shadows primarily emphasize the gathering and analysis of historical data, events and behaviors to offer insights and support decision-making. In contrast, digital twins are typically real-time virtual duplicates of real-world entities, capable of seamlessly synchronizing data with their real-world counterparts. Through a comprehensive analysis of the inherent distinctions between digital twins and digital shadows, we can enhance their effectiveness and relevance across various domains. This comprehension holds paramount importance in the advancement and implementation of digital twin technology.

**Figure 1:**
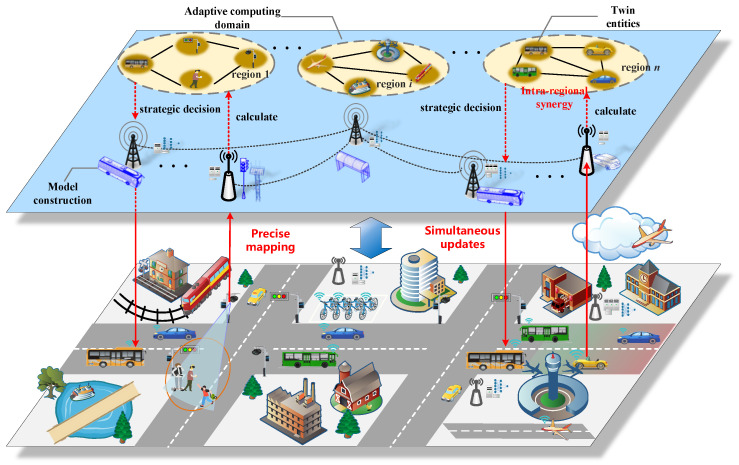
The illustration of a digital twin network in which complex tasks can be conducted through the cooperation among twin entities.

As the concept of the digital twin is further clarified, its application has begun to attract researchers’ attention. Zhao et al. [10] bring digital twin into the in-vehicle network to improve data collecting, prediction, verification and inspection capabilities. Wu et al. [4] defined a digital twin network (DTN) as a many-to-many mapping network comprised of numerous digital twins (DTs), in which physical entites and virtual counterparts can communicate with each other and cooperate to perform complex tasks. As shown in Figure 1, a digital twin network of a physical space is constructed in information space, and the twin entity selects a target base station for migration based on its physical entity’s real-time location to maintain a continuous low-latency information exchange with the physical entity. Furthermore, the physical entity and its corresponding twin entity maintain state synchronisation through real-time data interaction. Twin entites in DTN communicate and collaborate with each other to optimize system decisions to regulate the behavior of physical entities. Simultaneously, twin entities in the relevant domain can learn from one another to achieve the autonomous evolution of the twin network. Obviously, the emergence of digital twin networks will bring people’s research on digital twin technology to a new level.

While there are currently successful instances of digital twin implementation, there remains a scarcity of publicly accessible implementation specifics. This scarcity of information makes it challenging to assess their efficacy, conduct meaningful comparisons and collectively advance the development of digital twin methodologies [11]. In order to promote the better development of digital twins, some researchers have reviewed the existing work from different perspectives. In reference [12], the authors synthesized the different views on digital twins in the existing literature to sort out the development needs of standardization frameworks. The authors of reference [13] summarize the basic characteristics of digital twins in existing research and review how to classify digital twins based on these characteristics. Mihai et al. [14] have described how enabling technologies can be used to enable digital twin services, using real-world examples, and have emphasized the significance of enabling technologies for the functioning of digital twin services. Qi et al. [15] combined with the five-dimensional digital model to sort out the enabling technology and tools of DT, and provide the general application direction and tool selection of enabling technology. Hu et al. [16] conduct a review of different definitions, models and the six pivotal enabling technologies related to digital twins. Moreover, they shed light on some of the prevailing challenges present in current research pertaining to digital twin modeling. Liu et al. [17] provide a comprehensive and in-depth review of digital twins from conceptual, technical and application perspectives. Zhang et al. [18] provide a comprehensive discussion of the core elements of digital twin technology, including the concept, evolution, application tools, platforms and intersection with related technologies.

However, this work focuses on the investigating individual stages of digital twin development from the perspective of standardization framework requirements, feature-based classification, enabling technologies and tools. This approach does not provide readers with a comprehensive perspective on digital twins. In order to enhance the transparency, systematic approach and overall quality of our review process, we have adopted the 27-item checklist from PRISMA 2020 as our guiding framework for this review [19]. We selected the highly relevant and high-quality literature using criteria such as correlation, citation frequency and impact factors for our evaluation. As described in Table 1, we conducted a comprehensive and systematic overview of digital twins. The specifics of the work are as follows. First, we look at data reliability from the perspective of data collection, processing and management. Then, we discuss various approaches to model development and updating. Next, we explore computational offloading within the twin network from several perspectives, with particular emphasis on the issues of twin state synchronisation and twin entity deployment within the twin network. At last, we also summarize the application of digital twins. This paper achieves the entire closed loop of the digital twin system, going from the physical to the virtual and back to the physical. It presents a systematic representation of the whole process starting from the digital twin data, through the model and twin network and finally up to the application.

The rest of the paper is structured as follows. Section 2 analyzes and summarizes the reliability problem of digital twin data. Section 3 introduces the construction and updating methods of the digital twin model. Section 4 provides a systematic overview of related work within the twin network. Section 5 lists the applications of digital twins in different scenarios. Finally, Section 6 and Section 7 discuss the challenges and future work and conclude the research.

## 2. Digital Twin Data

Real-time information interaction between the physical and virtual worlds is the foundation for the accurate mapping of physical entities in the digital twin network. Thus, a detailed survey on the work related with digital twin data has been carried out in this section, including accurate data collection, data fusion and data computation and analysis. In addition, due to the importance of data reliability in the construction of high-fidelity models, the work on enhancing data security and privacy in digital twins has also been discussed.

### 2.1. Data Collection and Processing for Digital Twins

#### 2.1.1. Reliable Data Collection

The data for the digital twin system must experience the stages of data collection, data processing and data analysis. The collected data primarily consists of static modeling data, dynamic interaction data and service knowledge data. Sensors are typically used to collect data in physical systems. Damage to the sensors during this process can result in incorrect data collection, affecting the following stage of the system or possibly the ultimate result. In this regard, Darvishi et al. [20] proposed a machine-learning-based general sensor verification architecture based on a collection of neural network estimators and classifiers. The estimators are virtual sensors for all unreliable sensors, whilst the classifiers are used for detection and isolation tasks to ensure that the data obtained by the digital twin is reliable. However, the paper does not detail the practical applications and specific circumstances of sensor failure prediction and does not provide a viable solution to the data shortage. As there were inadequate data, Tao et al. [21] predicted the data by creating a digital twin of the device. This method efficiently solves the problem of data collection when there are inadequate sensors or when there is a failure. Furthermore, the artificial-neural-network-based digital model may mimic virtual twin data to compensate for the lack of real-world data [22].

Current research in data acquisition and processing has focused on ensuring data reliability by detecting sensor defects and developing digital twin models of devices to correct for data shortcomings, which can be accomplished with good results for conventional devices. However, for large and sophisticated systems, there may be a combination of insufficient data and difficulty in deploying sensors, otherwise building a twin system requires a large amount of reliable data to provide support. The previous approach does not facilitate data collection in this case, and, in the future, a modest amount of data coupled with expert knowledge could be used to develop a digital twin model.

#### 2.1.2. Data Fusion

The digital twin offers a variety of features such as visualization, real data simulation and performance monitoring. Some or all of these features can be implemented using existing systems that store product data. However, to construct a digital twin, this data must be brought together from multiple platforms. In addition, the same event may have different types of data from different sources, such as audio, video or video from different angles, all of which need to be fused. In response, Ala-Laurinaho et al. [23] created a data link system that provides a single interface via an application programming interface (API) gateway to merge data from numerous systems, allowing all data from a physical product to be accessible and available in a single location. However, the API gateway adds a degree of latency that is less suitable for control and monitoring applications that require low latency. The authors of reference [24] offer a digital twin data collaboration architecture that combines multidimensional and multi-state data into a data collaboration framework. To eliminate data silos, they model integration using precise data fusion relationships, connect data and relational databases and return results in JSON format via web requests. The above work provides a fresh perspective on data fusion for digital twin systems, but overlooks the heterogeneity of data at the various device levels. Bellavista et al. [25] proposed a method to handle the heterogeneity of industrial devices using Application-Driven Digital Twin Network (ADTN) middleware to address the issue of data heterogeneity.

The data fusion work described above enables the successful merging of heterogeneous data from many sources and data types. However, the fusion process does not take into account the ultra-low latency requirements of the digital twin system, which may cause deviation in the state synchronization between physical and virtual spaces.

#### 2.1.3. Analysis of Data Operations

The necessity to collect significant volumes of data from multiple devices in real time brings a data heterogeneity problem for digital twin systems, and the continuous and massive amount of data in real time poses a huge challenge to the system’s computing capability. Traditional centralized cloud computing can meet the computing capacity requirements, but the large distances to the device side do not meet the distributed and low latency requirements of digital twin systems. Mobile Edge Computing (MEC) offers a new solution to the this problem. Yang et al. [26] used an edge computing system to perform processing operations at the endpoint to reduce the computational load and data transmission delay in the cloud to meet the requirements of the digital twin system. But the collaboration between the edge and the cloud has not been considered. In response, Costantini et al. [27] proposed an IoTwins platform that collaborates on data processing and analytics by exploiting edge and cloud computing capabilities. In this situation, edge computing provides lightweight data computation and rapid response capabilities, especially for handling real-time data streams, whereas cloud computing supports large-scale simulations and machine learning algorithm training. However, the paper does not go into detail on how the IoTwins platform handles massive amounts of data or its data visualization capabilities. Ma et al. [28] proposed a digital twin test architecture for real-time data parsing, processing, visualization and analysis using two open source technologies, Thingsboard and Kafka, via a Faust agent to push data to a Kafka proxy server and Kafka consumers to provide various functions. The architecture allows users to visually analyze and display large amounts of production data.

### 2.2. Digital Twin Data Management

For a sophisicated digital twin system, twin data needs to be stored, accessed, shared and analyzed by many participants, which poses significant reliability risks to twin data. Traditional centralized data management facilitates security, confidentiality, consistency and reliability, but hacking or accidental damage to a single data center can result in data loss and irrecoverability, severely impacting the business. As a result, distributed data management is becoming increasingly popular, which can eliminate single points of failure and reduce data management costs and risks. However, in this case, vast amounts of data may be maintained by various stakeholders, and mistrust between different stakeholders may impede data sharing, placing greater demands on data security and privacy. Thus, we summarize data management challenges from three perspectives: data security, data privacy and data trustworthiness.

#### 2.2.1. Data Security

Data are at the heart of what drives the digital twin system, and ensuring data security in the data exchange process is critical. Data sharing between stakeholders and platforms necessitates accessing and extracting data from many databases, which can result in cyber-attacks [29] and data tampering during data storage, access and extraction, and cannot adequately ensure data security. To enable secure data sharing, the authors of reference [30] present a DT security architecture that tackles the security of data sharing via a digital twin adversary model. However, this solution necessitates model synchronization and does not address data security issues from the source. The authors of reference [31] propose a blockchain-based strategy for managing product digital twins by establishing a peer-to-peer network, which ensures that users securely communicate product lifecycle data over the network. However, there are difficulties with security and confidentiality in the data exchange process. The authors of [32] propose a blockchain-based information management system that addresses trust and security issues in digital twin data sharing by utilizing blockchain smart contracts to provide monolithic and multiple authentication to achieve trust in a decentralized manner, avoiding reliance on a central authority. To ensure data integrity while ensuring data sharing security, the authors of reference [33] propose a scheme that includes security features such as authentication, key negotiation, digital twin data storage in cloud servers and data hashing in the blockchain. This approach is effective in ensuring data security and integrity and is feasible and efficient in a digital twin environment.

The application of blockchain to the data storage and extraction process can effectively ensure the security and integrity of the twin data, however, the extraction process is complex and there may be latency pressures on the system during the extraction process. In addition, there may be trust issues in the storage and extraction of data from participants who provide unreliable data.

#### 2.2.2. Data Privacy

Privacy protection of data from various sources is required to prevent data leakage to the prejudice of the providers due to the multi-source nature of data providers in the interaction of digital twin systems. Federated learning is viewed as a potential option in the field of Industrial IoT to enable distributed data processing and learning while preserving privacy. To enhance privacy protection, some researchers are currently using distributed data processing and learning through federated learning [34]. To avoid disclosure of user information, Lu et al. [35] presented a strategy that combines blockchain with federated learning to reduce the risk of privacy leakage by training locally on the user side and only transfering model parameters. According to the research, current privacy implementations focus on using federated learning to build digital twin models of devices and avoid raw data transmission to enhance privacy protection [36,37].

#### 2.2.3. Data Credibility

Data reliability is an important consideration in the process of interacting with digital twins. Inaccurate data, especially for vital systems, can lead to poor decisions that result in severe financial losses. Thus, how to increase the confidence of digital twin data has become the most critical topic. The authors of reference [38] proposed to use blockchain to regulate the behaviour of participants and to secure data transmission and transactions. As the issue of data source dependability has not been addressed in digital twin technology, Suhail et al. [39] proposed an architecture that uses blockchain and digital twin technology in tandem. Blockchain is employed in this framework for secure data management, and digital twins are used to collect data from many sources and check for physical and virtual data discrepancies to improve data trust and security in critical infrastructure. Further work by the authors considers the data trust problem caused by uncertainty in the data generation and transmission process, and proposes a blockchain-based framework for the Industrial Internet of Things (IIoT). To ensure the trustworthiness of the data and the source of data generation, the source data is tracked and traced, starting from the primary objective of ensuring the reliability of the data source [40]. However, once a device is recognized as trustworthy on the network, any data can be exchanged with the device without further investigation. This is not a long-term guarantee of data security, and if an error occurs in the data, the trust issue will resurface and grow in importance. To address this problem, Ridhawi et al. [41] propose a new architecture that integrates a zero trust architecture into a digital twin-enabled 6G network to ensure the security, privacy and authenticity of physical devices and their digital twin counterparts via the continuous trust assessment of threat intelligence and decentralized authentication facilitated by blockchain. High levels of machine learning dispersion and generality are required to achieve decentralized zero trust. While meta-learning adds generality to learning models, it cannot be applied to IoT endpoints, and more research is required to achieve decentralized zero trust.

Currently, data trustworthiness research can ensure the trustworthiness of data at source, and continuous trust assessment and identity verification can be achieved through a zero-trust architecture to ensure the continuous trustworthiness of data. However, continuous verification and assessment can place additional computational pressure on the system, and the frequency of assessment and verification must be set appropriately to maximize resource efficiency. Furthermore, decentralized zero-trust has not yet been achieved, and the approach has limitations in terms of high levels of machine learning and generalizability that need to be addressed.

## 3. Construction of the Digital Twin

The realization of digital twin is mainly through the establishment of digital mirrors (called twin entities) for physical entities, through the integration and fusion of geometry, physics, behaviour and rules of the four-layer model, so that the each twin entity has the functions of evaluation, optimization, prediction, evaluation and other functions. Therefore, the modeling of twin entity is the core component of digital twin technology.

### 3.1. Modelling of Twins

The modelling of a twin entity, as a key component of the digital twin, must meet the high fidelity criteria of the digital twin, ensuring high accuracy and real-time performance to accurately represent the true state of the physical entity. Traditional modeling methods often rely on design numerical parameters for digital modeling, which may result in a large number of design iterations. With the introduction of digital twin technology, a new way of thinking about modeling methodologies has emerged. To solve the problem of model accuracy and loss of immediacy caused by data reliance on user feedback, Luo et al. [42] proposed a digital twin modeling strategy to achieve self-awareness, self-prediction and self-maintenance through digital twin description models, digital twin mapping models and digital twin intelligent models. To solve the problem of modeling the complex dynamics of aircraft, Mo et al. [43] created an intelligent system to model the complex dynamics of the physical world through real-time multimodal sensors and data inputs, which can be updated in real time to more accurately reflect the real state of physical entities. However, there are still problems with the amount and quality of data available for data-driven modeling, which limits the accuracy of the models [44]. In this regard, Wang et al. [45] proposed a data-driven dynamic modeling technique that uses data, models and algorithms to build virtual models and employs deep learning algorithms to process large amounts of data to improve data quality and ensure model accuracy.

In addition to data-driven twin modeling for physical entities, it is necessary to construct the model for the mutual relationship or interaction between them, which can help accurately depict the dynamic behavior of physical entities in digital twin systems. In reference [46], Guerra et al. used digital twins to model the behaviour of an ultra-precise motion system with gaps and friction. To address the problem of vehicle trajectory recovery, Ji et al. [47] used the proposed Spatio-Temporal Tracker (STT) to model the interaction between vehicles and their global motion patterns in IoV to capture dynamic and complex traffic situations to aid vehicle trajectory recovery. Liu et al. [48] developed a multi-scale quality knowledge model development technique based on digital twin to investigate product quality during processing. The multi-scale expression of product quality elements and the research of the link between quality indicators are realized by developing the quality knowledge map. Considering the multidimensionality of product data in the processing process, Liu et al. [49] propose a digital twin modeling method based on biosimulation, which uses biological simulation to merge multi-dimensional context machining process data to achieve a high fidelity and adaptability of the digital twin system as well as increase component processing accuracy and efficiency.

Current modeling methods allow the creation of digital twin models for multiple devices in multiple scenarios. However, there is no universal model construction method, and there are still issues with slow modelling speed and simulation lag; further research is needed to improve modeling speed and accuracy. Furthermore, by encapsulating models, model calls can be repeated quickly and cost-effectively, but there is no common model library to manage encapsulated models. Another research path in this area is to accomplish model invocation through quick model reconstruction. However, there is no appropriate solution for fast model reconstruction that supports fast model reconstruction while keeping accuracy, cost and energy usage in mind.

### 3.2. Update of Twin Model

The twin model must be updated regularly during the use of the digital twin model to ensure that it does not become outdated, to reduce the gap between the model and the physical entity and to reduce the synchronization effort once the model is re-created.

Current research on twin model updating has focused on updating models with actual or simulated parameters obtained from physical entities or virtual models. Kang et al. [50] proposed a model updating method in which the internal parameters of the model are obtained firstly via a simulation method, and then the model is updated using actual data output from a small number of physical entities to build a more accurate and reliable model. Since the internal parameters are not actual data, this method cannot ensure the accuracy of the model update. To address this issue, Jafari et al. [51] used a rolling learning technique to update the model parameters, where the model is retrained and updated on a regular basis with real-time data from the physical device to account for aging effects and preserve model correctness. However, the optimal model update frequency for continuous model modification requires further investigation. Kapteyn et al. [52] present a digital twin perception technique that uses a combination of physical models and machine learning classifiers. This method trains an interpreter using the optimal decision tree and updates the digital twin model by mimicking training data generated by physical entities in various states.

Due to the many perturbations that occur during device operation, the uncertainty of model updates rises, which makes it more difficult to update the model to reduce the deviation from the physical entity. In this regard, Tripura et al. [53] provide a framework for creating and maintaining digital twins of dynamic systems based on physics-based libraries. Using input and output information from system engineering or output observation, the candidate function library representing some physics is used to infer the new perturbation term in the existing digital twin model, and sparse Bayesian regression is used to fine-tune the correct perturbation term for model updating. According to msimulation results, this method can effectively infer new perturbation terms in the digital twin model, provides accurate and interpretable perturbation descriptions, and has a high model update performance.

Existing research efforts mostly focus on updating the model using model simulation data or physical entity data, and it can also identify uncertainty in the quantitative model update process to increase the accuracy of model update. However, if there is insufficient data in system engineering, the model simulation approach cannot ensure the accuracy of the simulated data. In addition, since the model update does not have high delay requirements, expert experience and historical data can be used to improve model accuracy.

## 4. Digital Twin Networks

**Figure 2:**
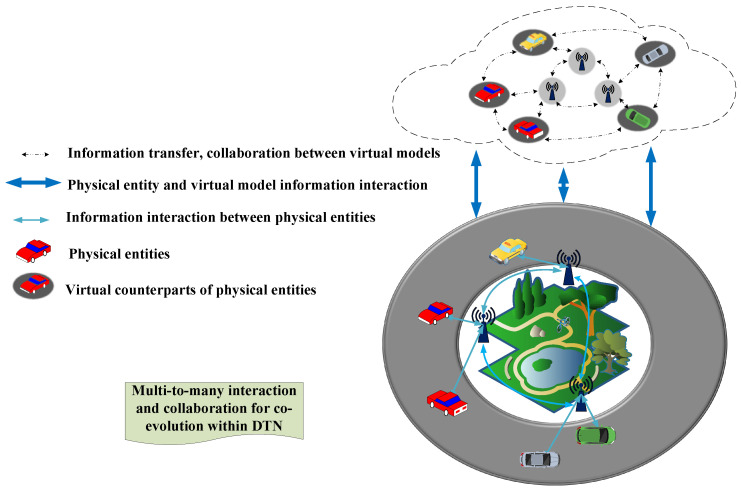
Digital twin network.

As application scenarios become increasingly complex, a single digital twin entity cannot meet the needs of the application, resulting in the emergence of twin systems or networks which are composed of many twin entities. In physical space, the communication and information exchange between many physical entities are required, thus the twins corresponding to physical entities also require information interaction, collaboration and mutual learning to perform complex system tasks. As shown in Figure 2, such multiple interconnected twins form an information exchange network, i.e. a digital twin network. In this chapter, we summarize the work on the implementation of physical–virtual state synchronization, both in terms of state synchronization and state correction, as well as twin deployment within twin networks and computational offloading.

### 4.1. Physical–Virtual State Synchronisation

To guarantee the high fidelity of the virtual model in digital twin systems, the state of the physical entity and the virtual model must be synchronized by real-time data interaction between the virtual model and the physical entity [54]. This allows the virtual device to simulate and forecast the actual device before it is used, resulting in more accurate data and analytic outcomes. Several synchronizing approaches have been presented in recent research to assure physical–virtual state synchronicity. In this section, the work on physical–virtual state synchronization is introduced, both in terms of state synchronization and state error correction.

#### 4.1.1. Synchronization of States

State synchronization is the real-time updating of the state of the virtual model in response to the real-time state of the physical entity in order to achieve the dynamic synchronization of the virtual model with the physical entity [55] and to ensure that the model accurately reflects the state of the physical entity. Since the models may exist at multi-disciplinary scales, synchronicity between multidisciplinary models must be considered in dual state synchronization. Talkhestani et al. [56] propose a synchronous approach for an interdisciplinary model, in which synchronization between digital twin models is achieved by determining the anchor points in each subsystem topology and matching the anchor points between different subsystems. This approach can detect changes in physical production systems and automatically solve multidisciplinary synchronization problems. However, it requires a centralized digital twin management system, which requires extensive planning and design. Considering the effect of data transmission rate on model synchronization during data interaction, Li et al. [57] present an event-driven model synchronisation technique based on digital twin systems. This technique achieves model state synchronization through the adaptive modification of transmission rate and data transfer on the basis of keeping the digital model’s response compatible with the physical system. However, due to poor data transmission and potential application delays, the real-time mapping of the twin model cannot be guaranteed.

If full synchronization cannot be achieved for the model state synchronization problem, it is possible to increase model accuracy by minimizing the synchronization time. Omar et al. [58] proposed an iterative approach to satisfy the model state synchronization requirement by reducing the average synchronization time between the physical and digital worlds through the optimal allocation of computational and communication resources. Similarly, Hashash et al. [59] developed a new edge-continuous learning strategy that takes into consideration the history-aware nature of DT and transforms the model updating process into a bi-objective optimization problem to minimize the event loss function and the corresponding desynchronization time. In response to the increasing desynchronization time, a regularized DT history elastic weight combining (EWC) technique is presented to reduce the desynchronization time.

Physical–virtual state synchrony ensures that the virtual model can properly monitor and predict the physical entities, but state synchronization requires accurate capture of real-time state information from the physical entities. Furthermore, how to synchronize data in physical and virtual space during data transfer. With the growing data size in the process of continuous state synchronization, model state update latency remains a challenge that needs to be addressed in the present research.

#### 4.1.2. State Error Correction

In practical applications, small inaccuracies in the twin model may occur due to the poor data capture of physical conditions, faulty modeling, time delays and parameter errors that may occur during the continuous updating of the model and model parameters. In this case, an error compensation method can be used to maintain the state synchronization of the twin. Cronrath et al. [60] developed a method based on reinforcement learning, which adjusts for residual errors using reinforcement learning and data fed back from the system. To reduce the gap between simulated and measured data, the authors of reference [61] bridged the relationship between virtual and physical space using an improved cycle GAN with smooth cyclic consistency loss. Considering the impact of bias on the system, the authors of reference [62] proposed a trust-based weighted aggregation method to mitigate the impact of digital twin bias by quantifying the contribution of devices to the federated learning global model. In addition, the authors further investigated and proposed a self-correction method for DT deviations that maintains the consistency of the physical–virtual state by self-correcting the empirical differences [63].

Current research focuses primarily on correcting the model after the error has been generated. However, how to dynamically compensate for errors, reduce model state synchronization delay and improve the accuracy of digital twins in the process of continuously updating models and model parameters will continue to be addressed.

### 4.2. Twins Deployment Optimization

The deployment of the digital twin entities must be considered throughout the implementation of the digital twin networks to ensure that any digital twin entity can interact with its corresponding physical entity in real time.

#### 4.2.1. Optimization of the Placement

Twin deployment requires the dynamic placement of twin entites depending on the mobility of physical entities, computing resources at the network edge, and low latency requirements between physical entities and their twins. Considering the limited processing resources of the edge cloud and the social relationships between IoT devices, Chukhno et al. [64] proposed an optimal solution to the network edge placement problem of socially connected IoT digital twins. The placement optimization problem was formulated as a mixed-integer linear programming model, and linear relaxation was used to convert it to a mixed-integer linear programming problem. To adapt to changing networks and provide users with high-quality services in real time, Lu et al. [65] proposed a deep-reinforcement-learning-based approach to find the optimal solution by considering the digital twin placement policy and the corresponding system latency. However, user mobility, edge server state and computational complexity pose challenges to the dynamic placement of twins. In this regard, the authors of reference [66] model the optimal placement problem for social digital twins as a quadratic allocation problem (QAP) and propose an approximation algorithm and two relaxation techniques to address the challenges of the deployment specifics, IoT device mobility and computational complexity of edge networks.

Current research in dynamic twin placement focuses on maintaining the connectivity of the physical–virtual counterpart and reducing latency. Good connectivity and low latency requirements can be met by transforming the placement problem into a linear mathematical problem for placement optimization. However, due to the dynamic nature of the network, the placement policy may only remain at the ideal placement for a limited amount of time, necessitating real-time updating to cope. Furthermore, questions of energy efficiency, sustainability and cost in twin placement will need to be addressed in the future.

#### 4.2.2. Migration of Twins

**Figure 3:**
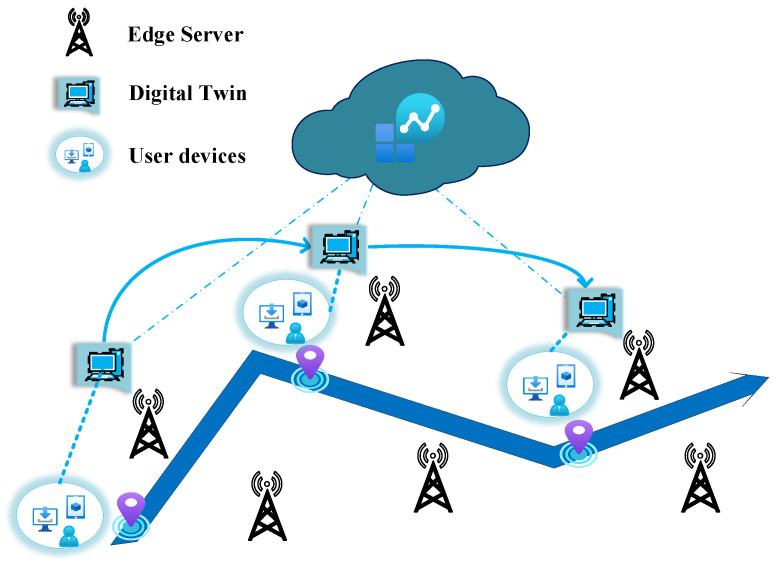
Digital twin migration.

Due to the mobility of physical entities, the twins need to be replaced frequently at a new server which is close to the current location of objects. The process of twin replacement is called twin migration, which is the transfer of the twin model from the source server to the target server to ensure efficient connectivity and low latency requirements for both physical and virtual counterparts. As shown in Figure 3, the digital twin follows the movement of the physical entity and migrates to a nearby target edge server. Up to now, the research on twin migration is very limited, and current work mainly focuses on identifying target servers and aggregating model parameters. Lu et al. [65] proposed a migration algorithm to perform twin migration. After identifying the target server, the method migrates the DT model parameters trained on the source server to the target server, and then transfers the experience samples related to the user’s device and updates the digital twin model with the new action and state space to achieve twin model migration.

The work above achieves good results by reconfiguring the twin model on the target server through migration learning, however, due to the high demand on computing resources, the digital twin migration process needs to consider the optimization of system load, storage and computing power. Furthermore, research is needed to improve the efficiency of twin migration, ensure the accuracy of twin migration and achieve seamless twin migration.

### 4.3. Computational Offloading within Twin Networks

For a complex digital twin application scenario, the digital twin network needs to undertake a large number of computational tasks, all of which have different time constraints and performance requirements. All the calculations and data exchange for the tasks ultimately require hardware where the twin entities are deployed to support. Due to the high complexity of tasks and the real-time requirements of twin systems, a single twin entity is clearly unable to undertake such tasks, thus requiring collaboration among multiple twins. Therefore, computional offloading in digital twin network is necessary.

#### 4.3.1. Single-User Calculation Offload

For computational offload in twin networks, researchers use digital twins to assist in optimizing communication links or offload strategies to improve user quality of service. In this regard, the authors introduce URLLC in the edge network to reduce the end-to-end (e2e) latency of compute-intensive tasks offloaded by multiple users by optimizing the communication link between IoT devices and access points (APs) [67]. However, as the number of users increases, intensive offload responses can overload servers and affect quality of service. In this regard, Dai et al. [68] investigated the impact of the number of users on server performance, proposed a digital twin network (DTN) to construct the network topology and the random task arrival model in the Industrial Internet of Things system. Similarly, the authors propose a method for joint communication and computation offloading with DT in a URLLC-based edge network, and propose an iterative algorithm based on an alternating optimization method and a convex approximation framework. The simulation results show that the algorithm can effectively reduce delay [69]. Furthermore, in order to meet the requirements of low-latency devices, Li et al. [70] propose the end-to-end (E2E) delay minimization problem of digital dual-assisted offload UAV-URLLC and introduce drones as mobile edge servers to assist communication while supporting real-time task offloading. The numerical results show that the proposed solution can solve the lowest latency problem with low power constraints.

**Figure 4:**
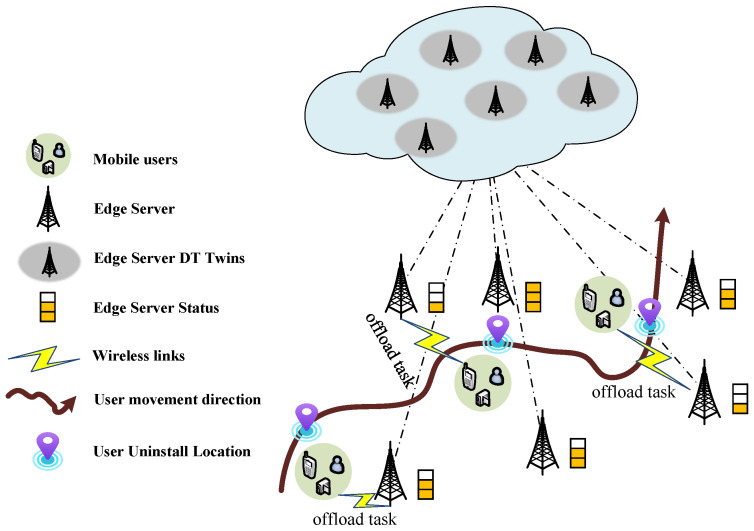
Mobile single-user calculation offloading.

In the case of offloading single-user computing from edge servers, this greatly increases the difficulty of offloading user computing due to the highly dynamic location of users. As shown in Figure 4, the user maps the state of the edge server through the digital twin during the moving process to select the target server for uninstallation and complete the task offloading during the move. To solve the above problems, Sun et al. [71] established a digital twin edge network (DITEN) to estimate the edge server state, and then used the Lyapunov optimization method to transform the long-term migration cost constraint into a multi-objective dynamic optimization problem, which was then solved using actor-critical deep reinforcement learning. The simulation results show that the scheme can effectively reduce the average offload delay, the uninstall failure rate and the service migration rate.

The above research can make full use of server computing resources and achieve user computing offloading from a single target server. However, since computing offloading considering the mobility of a single user requires a dynamic implementation of the MES selection and offloading process, there are high requirements on the system computing power. In addition, when selecting the target edge server for digital twin-assisted offloading, the accuracy of the edge server digital twin must be guaranteed to ensure the accuracy of the decision.

#### 4.3.2. Multi-User Calculation Offload

**Figure 5:**
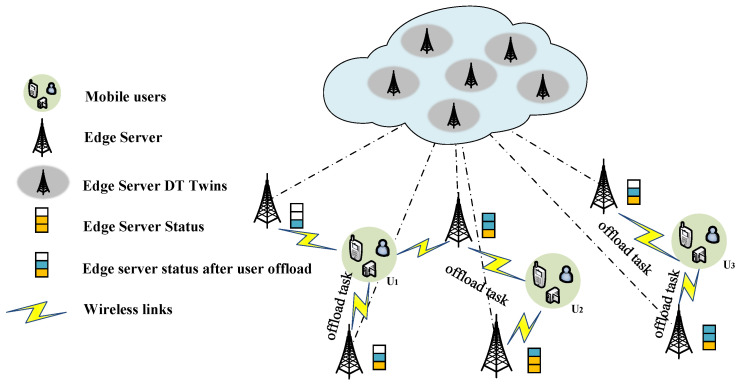
Multi-user task uninstallation.

In a twin network, the devices that need to compute offload are typically multiple in parallel and randomly distributed within a region. Based on the twin network, you can accurately understand the distribution of computing resources around you, so you can quickly and accurately distribute users’ computing tasks across multiple servers to reduce idle computing resources. As shown in Figure 5, users in multiple regions select multiple target servers for task offloading based on server status and their own task offloading requirements. To address how to intelligently offload multiple IoT device offload tasks to multiple mobile edge servers, the authors propose a mobile edge computing architecture based on digital twin technology, achieving end-to-end latency minimization within the constraints of computational resources and quality of service [72]. However, existing work ignores edge collaboration that can provide additional performance gains to the system. To enable a single user to intelligently offload tasks to multiple collaborative Mobile Edge Servers (MES) with the assistance of DT, Liu et al. [73] developed a DT-assisted task offloading system that uses channel state information (CSI) and blockchain to perform MES selection, and models the MU offloading process as a Markov decision process (MDP). The simulation results show that the strategy can ensure data security and edge server dependability, while drastically reducing the system’s power consumption and communication time.

In real-world large-scale scenarios, the uncertainty, dynamics and complex topology of user devices increase the difficulty of offloading computation. To ensure that the processing capabilities of each edge server are fully utilised in the multi-user mobile offloading situation, Xu et al. [74] proposed a DQN-based dynamic service offloading method for edge computing. This method establishes a multi-user computational offloading system model through digital twin, and combines the deep Q-network with experience playback and target network to optimize the offloading strategy. In order to meet the challenges posed by the heterogeneity of vehicles, by different applications with different requirements and by unpredictable vehicle topologies, Zhang et al. [75] introduced digital twin and artificial intelligence technology to the vehicle edge network, and proposed a coordinated graph-driven IoV task offloading scheme. The numerical results show that the system minimizes offloading costs under stringent network latency requirements compared to the benchmark technique.

The above research can use the communication between twins within the twin network to understand the offload requirements and server status of the users in scope, improving resource utilization by optimizing the offload policy so that the server maintains the maximum number of processing tasks under performance limits. This method can optimize the quality of user service while taking into account server operating costs or power consumption. However, the accuracy of the physical entity corresponding to twins in the twin network cannot be guaranteed, which may render the optimization uninstall strategy ineffective.

## 5. Digital Twin Applications

In this chapter, we will summarize the application of digital twins based on the following five scenarios: industrial environment monitoring and prediction, medical health monitoring and prediction, battery health management, building information management and traffic monitoring and prediction.

### 5.1. Monitoring and Forecasting of the Industrial Environment

In an industrial environment, the monitoring and predictive capabilities of digital twins provide a solution for determining the best maintenance strategy for equipment. To achieve reliable predictive maintenance of CNC machine tools (CNCMT), Luo et al. [76] proposed a CNCMT hybrid predictive maintenance method driven by DT. Booyse et al. [77] offer a predictive maintenance and system health monitoring strategy based on deep digital twin (DDT) technology to avoid relying on past failure data and case-by-case feature engineering. Furthermore, Dang et al. [78] proposed a digital twin framework based on cloud computing and deep learning to realize the continuous monitoring and active maintenance of structures. Liu et al. [79] proposed a data super-network fault prediction model and maintenance approach based on digital twins to enable fault prediction and the maintenance of mechanical products.

### 5.2. Healthcare Monitoring and Forecasting

The combination of digital twin and medical treatment provides new solutions for patients’ medical diagnosis, personal health monitoring and prediction. To realize personal health management in the whole life cycle of patients, Liu et al. [80] proposed a cloud medical system framework based on digital twin medical (Cloud DTH). Tai et al. [81] developed a digital bifunctional medical Internet of Things (IoMT) system for telemedicine simulation. The system can predict pathology in patients with mixed data (including redundant data) with over 90% accuracy, according to experimental results. In addition, aiming at the problem of medical resource investment under traffic restrictions, Lv et al. [82] introduced artificial intelligence algorithms, such as DL, to establish a UAV DTs information prediction model based on improved AlexNet to achieve the rapid and accurate delivery of medical resources.

### 5.3. Battery Health Management

Battery management is essential to improve the safety, reliability and performance of battery systems. In order to achieve efficient, accurate and visual battery management, Li et al. [83] proposed a cloud battery management system (BMS) to build a digital twin of the battery system, which enables the continuous and accurate monitoring of battery status. Considering the influence of battery materials, systems and the operating environment on battery life, Jafari et al. [51] proposed a structure of a digital dual-base battery for electronic vehicles, and predicted the state estimation of electric vehicle batteries using the limit gradient boost (XGBoost) model and extended Kalman filter (EKF), and responded to the battery state by electrocardiogram.

### 5.4. Building Information Management

At the construction level, digital twins are mainly used in the development and maintenance phases of construction projects. Aiming at the complexity and human error in the construction project management process, Pan et al. [84] combined building information modeling (BIM), data mining and Internet of Things to propose a closed-loop digital twin framework. In addition, the digital twin is also used for maintenance work after the project design has been completed. Lu et al. [85] proposed a DTs system architecture specifically designed at the architectural and urban level. Based on this architecture, a DT demonstrator is developed that integrates heterogeneous data sources to support effective data query and analysis, operation and maintenance management decision making.

### 5.5. Traffic Monitoring and Forecasting

At the realization level of the urban digital twin, traffic is a critical link in urban management and it is important to achieve traffic monitoring and prediction through the digital twin. In this regard, Sanchez-Vaquerizo et al. [86] proposed a new agent-based large-scale traffic microsimulation of the Barcelona urban area. In traffic scheduling, the problem of traffic data sparseness caused by IoV sensor data loss hinders scientific traffic scheduling decision making; Hu et al. [87] propose a short-term traffic flow and speed prediction method, TFVPtime-LSH. The feasibility of the proposed method in short-term traffic prediction is verified through experiments with real data sets.

## 6. Research Challenges

### 6.1. Data Filtering

The processing of massive amounts of real-time data by the digital twin system presents a challenge to its computing resources. To reduce the system’s computational burden, potential solutions include optimizing the computing offloading strategy or filtering high-value data from the larger dataset.

### 6.2. Reconstructing Digital Twins

The timely deployment of the digital twin system depends on the quick reconstruction of the digital twin of the corresponding physical entity in the system, and current researchers mainly focus on the construction of the twin model and rarely consider the reusability of the twin model. Furthermore, the costs of deploying the twin model in practical applications and its accuracy present challenges for achieving the swift reconstruction of the twin.

### 6.3. Migration of Twin Entities

In the case of twin migration, the current study cannot guarantee that the model will be continuously updated during the migration process, which poses new challenges for maintaining physical–virtual state synchronization during model migration. In the future, research should aim to enhance model migration efficiency while ensuring model accuracy, to reduce migration time and minimize the physical–virtual state bias caused by migration.

### 6.4. Splitting Digital Twins

Random resource distribution in the real environment may result in inadequate resource allocation for placing digital twins in various regions. To ensure the normal operation of the digital twin, it may be considered to split the digital twin of the same physical entity into multiple sub-digital twins and distribute them to a nearby resource-rich area.

### 6.5. Evolution of the Digital Twin

The evolution of the digital twin refers to the overall performance improvement of the digital twin system in the twin network as a result of information interaction and self-learning between numerous pairs of DTs within the DTN. However, the creation of digital twins requires the blessing of numerous computing, communication, security, privacy and artificial intelligence technologies, and future researchers will need to conduct more extensive research in this field to bring this idea to fruition.

## 7. Conclusions

In this paper, we provide a comprehensive and systematic overview of existing work on digital twins in four areas: data, models, networks and applications. First, we discuss the reliable handling and management of twin data in terms of data reliability, and then we summarize the different approaches to twin modeling and updating. In particular, we focus on the problems of twin state synchronization and twin deployment within twin networks, and computational offloading within twin networks from different perspectives. Furthermore, we provide a summary of the applications of digital twins. Finally, we consider the research challenges and future directions of digital twins, including data management, twin construction and the evolution of digital twins, in the hope of providing some ideas for further research in this area.

## Figures and Tables

**Table 1:** Comparison of characteristics in reviews.

Reference	Definition	Fundamental Characteristics	Applications	Twin Networks	Models	Data
[12]		✓	✓			
[13]	✓	✓	✓			
[14]			✓			
[15]			✓		✓	✓
[16]	✓				✓	
[17]	✓		✓			✓
[18]	✓					
Our paper	✓	✓	✓	✓	✓	✓

## Data Availability

Not applicable.
